# Implementation and Evaluation of a Newborn Hearing Screening Database in a Resource-Limited Setting: Advantages and Limitations

**DOI:** 10.3390/children13010022

**Published:** 2025-12-22

**Authors:** Krittipong Parangrit, Jutatip Sillabutra, Suwicha Kaewsiri Isaradisaikul, Kanokwan Kulprachakarn

**Affiliations:** 1 School of Health Sciences Research, Research Institute for Health Sciences, Chiang Mai University, Chiang Mai 50200, Thailand; krittipong_p@cmu.ac.th; 2 Otolaryngology Unit, Chiangrai Prachanukroh Hospital, Chiang Rai 57000, Thailand; 3 Department of Biostatistics, Faculty of Public Health, Mahidol University, Bangkok 10400, Thailand; jutatip.sil@mahidol.ac.th; 4 Department of Otolaryngology, Faculty of Medicine, Chiang Mai University, Chiang Mai 50200, Thailand

**Keywords:** database integration, digital health system, newborn hearing screening, resource-limited settings

## Abstract

**Highlights:**

**What are the main findings?**

• Newborn hearing screening database improved coverage, referrals, and ABR timeliness.
• Enhanced workflow efficiency but limited by data completeness and connectivity with other health information systems.

**What are the implications of the main findings?**

• Digital database integration strengthens early hearing detection in low-resource set-tings.
• Interoperability and staff training are key for sustainable implementation.

**Abstract:**

**Background:** Congenital hearing loss affects 1–3 per 1000 newborns and requires early detection to prevent developmental delays. Although Thailand implements universal screening, fragmented data systems limit effectiveness. To address this, Chiangrai Prachanukroh Hospital introduced a dedicated newborn hearing screening (NHS) database in 2023 to improve tracking, coordination, and monitoring in a resource-limited setting. **Objectives:** To evaluate the advantages and limitations of NHS database integration on screening coverage, referral rates, follow-up completion, and diagnostic timeliness. **Methods:** A retrospective analytic study was conducted over 24 months, comparing outcomes before (July 2022–June 2023) and after (July 2023–June 2024) database implementation. Key indicators included screening coverage, follow-up attendance, diagnostic ABR completion, and workflow efficiency, with the study period also encompassing the implementation of the database and adaptations to the screening algorithm. Data were analyzed using the chi-square test and fisher’s exact tests, supplemented by qualitative observations of system performance. **Results:** Among 8290 newborns, screening coverage before one month increased from 83.47% to 96.64% (*p* < 0.001), while referral rates decreased from 18.44% to 6.47% (*p* < 0.001). Diagnostic ABR completion improved from 7.41% to 52.63% within three months (*p* < 0.001) and from 59.26% to 84.21% within six months (*p* = 0.06). The database improved workflow coordination, but challenges persisted, including incomplete data, limited interoperability, caregiver-related follow-up barriers, and low hearing-aid uptake. **Conclusions:** Integration of the NHS database, as well as protocol changes, improved screening coverage, referral accuracy, and diagnostic timeliness, but follow-up and early intervention barriers persisted. Continued progress will require stronger interoperability, improved family engagement, and digital infrastructure investment, with tele-audiology and decision-support tools helping expand access and efficiency.

## 1. Introduction

Congenital hearing loss, one of the most common sensory disorders in newborns, has an estimated global incidence of 1–3 per 1000 live births, with Thailand reporting a similar prevalence of approximately 1.7 per 1000 live births [1,2]. The consequences of undiagnosed or delayed identification of hearing loss in newborns are profound and enduring. Children with unrecognized hearing impairment are at heightened risk for delayed speech and language development, impaired cognitive skills, reduced academic achievement, and long-term social and emotional difficulties [3,4]. These outcomes are often preventable if hearing loss is detected and managed early in life. In response, many countries have adopted the Early Hearing Detection and Intervention (EHDI) framework, a structured public health approach designed to identify and manage congenital hearing loss during the first months of life. Over the past decade, several low- and middle-income countries (LMICs), including Thailand, have made substantial strides in expanding Universal Newborn Hearing Screening (UNHS) programs. Thailand has implemented a national UNHS program since 2021; however, national performance indicators remain variable across regions. National reports indicate that screening coverage typically ranges from 93.10% to 99.78%, with referral rates between 2.51% and 14.49%, and diagnostic completion within three months ranges from 20.13 to 90.91%, particularly in public hospitals with limited audiology resources [5,6,7]. Hearing-aid fitting before six months of age is similarly uncommon, reflecting systemic barriers to early intervention. These efforts have been supported by national policies and structured pilot programs under the Ministry of Public Health, which have promoted NHS implementation and digital reporting systems across multiple provinces [8,9]. However, the implementation of UNHS in LMIC settings continues to face critical systemic and operational challenges. Additionally, Thailand’s national NHS algorithm underwent a major revision in July 2023, changing from screening within seven days and rescreening at three months to screening within 24–48 h after birth and rescreening at one month. A major barrier lies in the limitations of data collection, tracking, and coordination mechanisms within healthcare institutions. In many resource-limited settings, including tertiary hospitals in Thailand, the absence of integrated digital systems has led to fragmented workflows for hearing screening programs. Prior to the introduction of a dedicated digital platform, Chiangrai Prachanukroh Hospital, a regional tertiary referral center in northern Thailand, relied on google forms and multiple manual spreadsheets, which were used across different units and screening periods to manage data. While accessible, these tools were not designed for longitudinal tracking or interdepartmental coordination. Screening results were often recorded in isolation and required extensive cross-checking for follow-up scheduling. Fragmented data across maternity, pediatric, and audiology services created delays and discrepancies in identifying newborns requiring rescreening or diagnostic ABR testing. The inability to generate automated alerts or unified follow-up lists increased the risk of missed cases. Moreover, the manual nature of reporting limited the hospital’s ability to monitor indicators such as screening coverage, referral rates, follow-up return rates, and age at diagnosis. To address these gaps, the hospital implemented a dedicated Newborn Hearing Screening (NHS) database in 2023. This standalone platform centralized data, supported structured entry, and generated follow-up lists, enabling semi-automated workflows and improved tracking. The transition represented a critical turning point, but the actual impact of database implementation on program performance remains insufficiently documented in LMICs. This study evaluated the advantages and limitations of NHS database adoption, focusing on coverage, referral, follow-up tracking, and diagnostic completion, while describing operational improvements and persisting challenges.

## 2. Materials and Methods

### 2.1. Study Design

A retrospective analytic study was conducted using 24 months of NHS data at Chiangrai Prachanukroh Hospital. Data were reviewed in two phases: before database integration (July 2022–June 2023) and after database integration (July 2023–June 2024). Newborns who died or were transferred before 1 month of age were excluded from both groups.

### 2.2. Screening Workflow and Database Integration

Before the introduction of the dedicated NHS database, the NHS workflow followed the ‘old’ system, which relied on google forms and multiple manual spreadsheets for data entry and tracking. During this pre-integration phase, staff entered demographic information, dates and locations of screening, laterality of testing, TEOAE results (pass/refer) (Sentiero device, Path medical, Germany), and basic risk-factor classification into google forms, which were subsequently exported into Microsoft Excel spreadsheets used as the primary data repository. The screening protocol followed a two-stage TEOAE process prior to audiology referral. Newborns received the first TEOAE screening before hospital discharge, performed by ward nurses. Newborns who passed were discharged from further screening. Those who referred on the first test underwent a second in-hospital TEOAE screening. Newborns who failed both screenings were scheduled for outpatient TEOAE rescreening at the Otolaryngology unit, where an audiologist conducted the assessment. Newborns who passed the outpatient rescreening were discharged, whereas those who continued to fail were referred for diagnostic evaluation, including auditory brainstem response (ABR) testing (SmartEP device, Intelligent Hearing Systems, Miami, FL, USA). The audiology team manually cross-checked records against paper files, prepared follow-up lists for rescreening or diagnostic ABR, and scheduled appointments by phone or handwritten notes.

In the post-integration phase, the program was purpose-built for NHS and developed through a national research initiative in Thailand, later adopted at Chiangrai Prachanukroh Hospital. The NHS database is a web-based platform integrated into the hospital intranet. Screening nurses enter OAE results at the point of care using structured data fields, including demographic information, screening result status, and next appointment dates, while automated completeness checks flag missing or inconsistent entries. For newborns requiring rescreening, the system automatically generates daily and weekly follow-up lists and alerts staff when repeat testing is due. Audiologists record diagnostic ABR results directly into the system, allowing real-time status updates. Automated functions include generation of follow-up lists, overdue alerts, and pre-allocated ABR appointment queues, although verification of caregiver attendance remains semi-manual. The platform demonstrated minimal downtime, and staff were trained through a brief one-hour session with supervised practice, allowing structured and reliable data capture to support program evaluation.

### 2.3. Statistical Analysis

Categorical differences between the two groups were analyzed in SPSS version 21, with statistical significance set at *p* < 0.05. Screening coverage, referral rates, and follow-up completion were evaluated using chi-square analysis, while diagnostic outcomes were assessed with Fisher’s exact procedure because of small cell sizes.

## 3. Results

**Figure 1:**
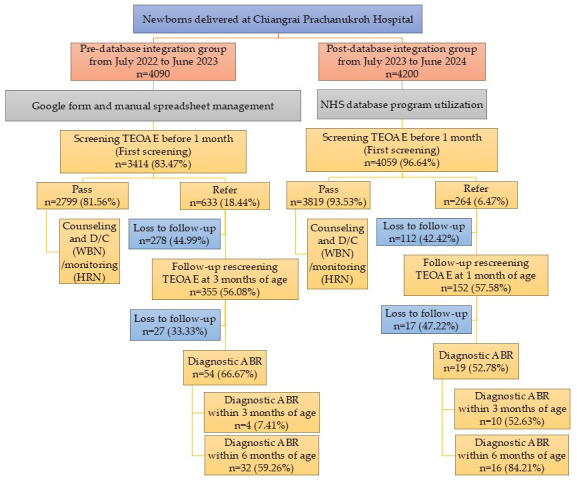
Flow of newborn hearing screening, follow-up, and diagnostic pathways between pre- and post- database integration. Abbreviation: TEOAE, transient evoked otoacoustic emissions; D/C, discharge; WBN, well-baby newborns; HRN, high-risk newborns; ABR, auditory brainstem response. Note: Denominators differ because “screening ≤ 1 month” includes only newborns screened within the first month, whereas pass/referral rates use the total number screened.

As shown in Figure 1, during the pre-database integration period, 4090 newborns were delivered, of whom 3414 (83.47%) underwent hearing screening before 1 month of age. Of these, 2799 (81.56%) newborns passed the initial TEOAE screening and 633 (18.44%) were referred. Among those referred, 278 (44.99%) newborns were lost to follow-up, while 355 (56.08%) attended rescreening, resulting in 54 newborns (66.67%) completing diagnostic ABR. Only 4 (7.41%) newborns achieved diagnostic confirmation within 3 months of age, and 32 (59.26%) by 6 months, as per the national recommendation. In comparison, 4200 newborns were delivered during the post-database integration period, and 4059 (96.64%) received screening before 1 month. A total of 3819 (93.53%) newborns passed the initial screen, whereas 264 (6.47%) were referred. Of those referred, 112 (42.42%) newborns were lost to follow-up and 152 (57.58%) underwent rescreening, leading to 19 (52.78%) newborns completing diagnostic ABR. Among these, 10 (52.63%) newborns were diagnosed by 3 months, meeting the JCIH benchmark, and 16 (84.21%) were diagnosed by 6 months, aligning with the national recommendation. Baseline demographics and clinical risk profiles were similar between cohorts, minimizing the likelihood that population differences contributed to the observed outcomes.

**Figure 2:**
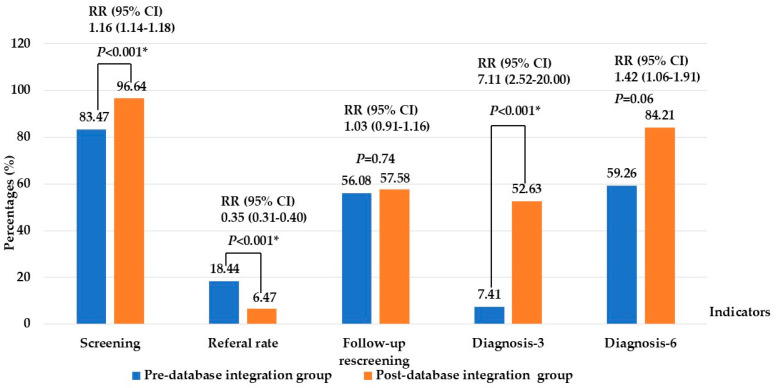
Comparison of key performance indicators of newborn hearing screening between pre- and post- database integration. Note: * Significant at *p*-value < 0.05; Screening: screening ≤ 1 month of age; Diagnosis-3 and -6: audiological diagnosis ≤ 3 and -6 months of age.

Quantitative comparisons showed significant improvements after database adoption. Screening coverage before 1 month of age increased from 83.47% to 96.64% (*p* < 0.001) (RR 1.16, 95% CI 1.14–1.18), while the referral rate decreased markedly from 18.44% to 6.47% (*p* < 0.001) (RR 0.35, 95% CI 0.31–0.40). Although overall follow-up completion rates did not differ significantly (56.08% vs. 57.58%) (*p* = 0.74) (RR 1.03, 95% CI 0.91–1.16), the proportion of infants completing diagnostic ABR by 3 months increased substantially from 7.41% to 52.63% (*p* < 0.001) (RR 7.11, 95% CI 2.52–20.22). Moreover, the proportion diagnosed by 6 months increased from 59.26% to 84.21% (*p* = 0.06) (RR 1.42, 95% CI 1.06–1.91), meeting the national target and aligning more closely with JCIH benchmarks. These findings demonstrate that database integration enhanced early diagnosis and alignment with JCIH benchmarks, as shown in Figure 2.

**Figure 3:**
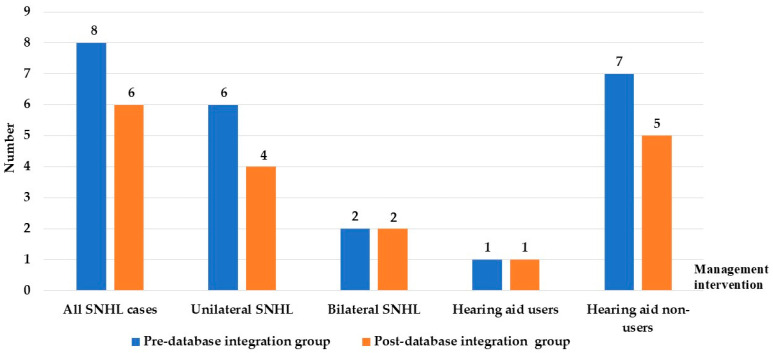
Distribution of SNHL cases and hearing aid management between pre- and post- database integration. Abbreviation: SNHL, sensorineural hearing loss.

Figure 3 shows that a total of 8 children were diagnosed with Sensorineural Hearing Loss (SNHL) in the pre-database integration group compared with 6 children in the post-database group. Unilateral SNHL was observed in 6 cases before integration and 4 cases after, whereas bilateral SNHL remained constant at 2 cases in each group. Hearing aid uptake was limited, with only 1 child in each group receiving amplification, both beyond the recommended 6-month intervention window. The majority of diagnosed children did not receive hearing aids, with 7 cases pre-integration and 5 cases post-integration. These findings indicate that while database integration improved the timeliness of diagnosis, challenges remain in ensuring timely hearing aid fitting and intervention for affected children, as shown in Figure 3.

## 4. Discussion

The present study provides one of the first real-world evaluations of hospital-level NHS database integration in a resource-limited setting in Thailand. The findings demonstrate that improvements were observed following database implementation. However, persistent challenges remain in follow-up completion, intervention uptake, and data quality, underscoring the complexity of sustaining EHDI benchmarks in LMICs. Program efficiency and screening outcomes demonstrated measurable improvements following database integration. Integration of the dedicated NHS database yielded clear benefits in workflow management. Screening coverage before one month of age increased statistically significantly from 83.47% to 96.64%, surpassing both national and international benchmarks. This improvement was largely attributable to enhanced workflow coordination and real-time tracking enabled by the NHS database, which reduced missed cases and supported timely screening before discharge [10,11]. However, because this study compared two consecutive 12-month periods without a control group, the observed differences may also reflect temporal changes unrelated to database implementation. Increasing staff experience, periodic refresher training, or concurrent public health activities could have contributed to improved performance across the screening pathway. Interpretation must also consider that Thailand’s national NHS algorithm underwent major revisions in July 2023, coinciding with database introduction. The shift from screening within 7 days and rescreening at 3 months to screening within 24–48 h and rescreening at 1 month likely contributed to improvements in first-screen timing. Thus, these outcomes represent the combined effect of national workflow changes and database supported enhancements rather than database impact alone. The referral rate decreased substantially from 18.44% to 6.47% following database implementation. This reduction reflects workflow improvements rather than changes in physiological screening performance, as the database minimized process related errors including incomplete documentation, missed second screenings, and premature refer entries, that previously inflated referral numbers [6,10]. The database did not alter OAE device thresholds or clinical screening protocols but standardized data entry, enabled real-time verification, and ensured that infants requiring repeat screening were correctly identified before being labeled as referrals. These workflow-driven improvements likely reduced false-positive referrals and enhanced accuracy across the screening pathway. Importantly, diagnostic confirmation by three months increased from 7.41% to 52.63%, while confirmation by six months reached 84.21%, approaching the JCIH “1-3-6” target [9,12]. However, interpretation of this improvement must consider concurrent protocol changes. In the post-integration period, the rescreening interval was shortened from approximately three months to one month, which facilitated earlier ABR referral; therefore, the observed gains cannot be attributed solely to database implementation. Interpretation of diagnostic timing also requires caution due to the small and unequal denominators between periods. In the post-database cohort, only 19 infants reached the diagnostic ABR stage, so each case accounted for roughly 5.3% of the overall proportion. As a result, the apparent increase—from four to ten infants diagnosed by three months—produces percentage estimates that are inherently unstable. The reduction in the number of infants requiring ABR (54 to 19) aligns with the markedly lower referral rate (18.44% to 6.47%) and likely reflects improved screening specificity and more efficient rescreening workflows. The analytic approach also imposed limitations. Diagnostic timing was dichotomized (≤3 months vs. >3 months) because assessment dates were recorded categorically rather than as continuous time intervals. Although aligned with JCIH benchmarks, this reduced granularity and may have obscured meaningful variation in diagnostic trajectories. More refined approaches, such as survival analysis or comparisons of median time-to-diagnosis, would yield greater interpretive value if individual-level time data were available. Additionally, multiple screening and diagnostic outcomes were evaluated, increasing the potential for inflated Type I error, although these outcomes represent sequential steps within a single clinical pathway. The NHS database primarily functioned as a workflow-enhancement tool rather than a diagnostic decision system. Its benefits stemmed from structured data entry, improved completeness, automated follow-up list generation, and reduced administrative errors, all of which contributed to more timely screening and diagnostic processes. User feedback indicated that the system was easy to operate after brief training and experienced minimal downtime, supporting feasibility in resource-limited settings. These observations reflect operational characteristics reported during implementation and are therefore presented as interpretative insights rather than quantitative outcomes. Follow-up tracking and diagnostic timeliness showed notable improvements after database integration. Although the overall follow-up completion rate changed only modestly (56.01% vs. 58.46%), the quality and timeliness of follow-up improved, supported by real-time alerts and pre-allocation of ABR diagnostic slots that facilitated earlier evaluation, particularly for high-risk newborns. Prior research at our hospital demonstrated that missed follow-up is largely driven by socioeconomic and geographic barriers, caregiver work demands, long travel distances, forgetting appointments, and low perceived importance of follow-up rather than system limitations [13]. These factors cannot be fully addressed by a database alone, which explains why improved tracking did not produce substantial gains in follow-up completion. Compared with other LMICs reports, in which follow-up loss often exceeds 30–40% [14,15], the relatively stable loss-to-follow-up rate in this study suggests that database integration minimized, but did not eliminate, systemic barriers such as caregiver awareness, transportation, and inter-hospital referral difficulties. International experiences, including the South Korean national database and Dutch regional coordination systems, similarly emphasize that digital integration is necessary but insufficient without concurrent investment in family-centered follow-up and outreach strategies [16,17]. Despite earlier diagnosis, persistent gaps in intervention were evident. Only one child in each cohort received hearing aids, both beyond six months of age, leaving most diagnosed infants untreated. This pattern mirrors other LMICs contexts, where financial constraints, limited device availability, and inadequate rehabilitation pathways delay intervention [18,19]. Review of individual cases showed that delays were primarily caregiver-driven; several families did not return for scheduled fitting appointments despite automated reminders and follow-up calls, resulting in prolonged time to amplification. These findings indicate that improvements in screening workflow alone cannot ensure timely intervention. Structural barriers, including caregiver readiness, financial concerns, logistical constraints, transportation difficulties, low parental awareness, and limited pediatric audiology resources, require targeted strategies beyond database implementation. Several benchmarks traditionally influenced by digital infrastructure, including accuracy of documentation, completeness of rescreening logs, referral tracking, and loss-to-follow-up showed smaller improvements. This pattern suggests that although the database strengthened internal coordination and reduced administrative errors, caregiver-level and socioeconomic factors remain the primary drivers of persistent follow-up challenges. Global EHDI experiences increasingly incorporate tele-audiology and mHealth solutions to address distance and workforce limitations. Tele-diagnostic infant assessments in South Dakota and rural South Africa have demonstrated that remote ABR services are feasible and can expand access for infants who fail initial screening [20]. Recent reviews indicate that remote hearing screening and intervention are non-inferior to traditional pathways and may reduce loss-to-follow-up [21]. mHealth-based hearing-health programs in LMICs further underscore scalable, low-cost approaches for caregiver engagement and follow-up support [22]. Finally, although significant differences were observed between the pre- and post-integration periods, this study was limited to univariate analyses. Incomplete or inconsistently recorded individual-level variables prevented multivariate modeling, limiting our ability to identify independent predictors of outcomes. As a before–after study without a control group, the observed improvements cannot be attributed solely to the database implementation. Changes in screening coverage, referral accuracy, and diagnostic workflow may also reflect temporal trends, increasing staff experience, or other concurrent operational adjustments. Therefore, the findings should be interpreted as correlational rather than causal, as the design does not allow for disentangling database effects from other influencing factors.

The findings highlight how digital transformation in screening workflows can enhance program efficiency, coordination, and diagnostic timeliness while revealing persistent challenges in sustainability and interoperability. The implementation of a dedicated NHS database produced measurable improvements across multiple dimensions of program performance, particularly in workflow efficiency, interdepartmental coordination, and data-driven monitoring. Integration of the NHS database markedly improved workflow efficiency and overall program performance across departments. The centralized data entry system minimized redundant paperwork and manual cross-checking errors, while real-time tracking of screening and follow-up processes allowed healthcare teams to promptly identify missed or delayed cases [23]. Although improvements in workflow efficiency were observed operationally during implementation, workflow efficiency itself was not directly measured as a quantitative outcome. Therefore, these observations should be interpreted as system-level insights rather than data-derived findings. This improvement reflects qualitative observations, as the number of data-entry errors was not quantitatively recorded. Automated scheduling of diagnostic ABR slots and parent reminders enhanced coordination between maternity, pediatric, and audiology units, thereby reducing staff workload and communication gaps. These features collectively facilitated earlier completion of both screening and diagnostic procedures and improved adherence to the JCIH “1-3-6” benchmarks [11]. The database also introduced standardized and structured data fields that strengthened data governance and monitoring capacity. Automatic generation of summary reports and performance dashboards enabled continuous monitoring of screening coverage, referral rates, and diagnostic completion, aligning with both national and international performance indicators. These real-time analytics provided hospital administrators with actionable insights for quality improvement. Similar outcomes have been reported from other digital EHDI platforms in South Korea, Israel, and the Netherlands, where centralized databases were shown to reduce referral rates and accelerate early diagnosis [10,16,17,24]. Unlike systems reported in South Korea or Israel, which primarily serve as centralized reporting tools, the Thai NHS database integrates real-time point-of-care data entry, automated follow-up generation, and diagnostic workflow coordination within a single platform. Its low-cost, intranet-based design and minimal training requirements make it particularly suited to resource-limited provincial hospitals, highlighting the system’s unique contribution to LMIC newborn hearing screening models. From a coordination perspective, the integrated referral module allowed seamless communication between primary, secondary, and tertiary hospitals through unified appointment management. Both sending and receiving facilities could track patient status and reschedule appointments instantly, reducing caregiver travel burden and improving follow-up compliance. Given this tiered structure, effective communication and data exchange across hospital levels are essential to support accurate referral pathways, timely rescreening, and completion of diagnostic follow-up for outborn and referred infants. These enhancements were based on qualitative workflow observations, as they were not measured quantitatively. The database supported more equitable access to diagnostic and rehabilitative services across regional networks by standardizing referral workflows and enabling shared, real-time tracking across facilities, thereby reducing geographic disparities and contributing to improved clinical timeliness for high-risk newborns. Furthermore, the system strengthened data governance through multi-level user authorization and encryption protocols that ensured confidentiality and accountability in accordance with Thailand’s PDPA. This level of information security represents a critical prerequisite for public trust and large-scale implementation of digital health systems in LMICs.

Despite these improvements, several operational and structural limitations remain. First, data accuracy still relied on staff compliance and manual entry, as automatic validation tools were not embedded in the system. This limitation occasionally resulted in incomplete or inconsistent data fields, reflecting the need for automated verification and standardized operating procedures to enhance data reliability. Second, interoperability with national health information systems and private hospital databases remains limited. The NHS database has not yet been linked to Thailand’s HDC, electronic claims system (e-Claim), or other Ministry of Public Health (MOPH) registries. Third, dependence on internet connectivity in rural hospitals posed operational constraints. Network instability delayed real-time data synchronization, particularly in mountainous or remote areas, while smaller hospitals with limited diagnostic resources were unable to fully utilize the system’s capabilities. Fourth, persistent loss to follow-up among outborn or high-risk newborns remains a challenge. This limited data exchange hindered long-term tracking of outborn newborns delivered outside the study hospital and referred from external facilities, who often lack unified electronic records and therefore account for a substantial proportion of loss-to-follow-up. Although real-time alerts and tracking improved follow-up for inborn newborns, incomplete inter-hospital data linkage continues to limit continuity of care. This pattern is consistent with global experiences, where even well-designed digital systems cannot fully overcome barriers related to transportation, parental awareness, or socioeconomic constraint [14,15,18,19]. Finally, compliance with PDPA presents ongoing challenges. Although the database incorporates encryption and controlled access, further refinement such as user certification, audit trails, and data anonymization, is needed to ensure full legal compliance and to support national-scale adoption.

For long-term sustainability, the NHS database should evolve toward interoperability with existing MOPH platforms such as the HDC, e-Claim, and other national registries. Seamless integration would enable longitudinal tracking of outborn newborns, reduce data fragmentation, and facilitate continuity of care across provinces. Linking the NHS system with maternal and child health records could also support holistic care pathways that connect hearing screening data with vaccination, growth monitoring, and developmental follow-up. Continuous training and technical support are vital to ensure consistent and accurate data entry, particularly in peripheral hospitals where staff turnover is high. Establishing regional “data stewards” or coordinators to oversee data quality and troubleshoot system issues can further strengthen accountability. From a policy perspective, incentive mechanisms such as pay-for-performance (P4P) schemes and recognition-based awards may enhance data accuracy and timeliness, as demonstrated in other digital health programs. Embedding automated error-checking, reminder notifications, and decision-support algorithms could also reduce human error and improve operational efficiency [25]. Integration of tele-audiology such as remote ABR interpretation or virtual counseling would expand access to diagnostic and rehabilitative services in geographically isolated areas [21,25]. Ensuring sustainability requires national-level investment in server maintenance, cybersecurity, and system upgrades. Budget allocation for these components should be incorporated into hospital operational plans and MOPH service frameworks. Collaboration with academic institutions and technology partners can drive continuous innovation while maintaining alignment with national policy priorities. Translating the benefits of database integration into sustainable national outcomes demands a comprehensive policy framework that emphasizes interoperability, human resource development, and continuous quality improvement across all levels of the healthcare system [26]. Ultimately, the NHS database represents a scalable model for digital transformation in early hearing detection and intervention programs in resource-limited settings. Its successful implementation in a provincial hospital demonstrates that structured digital systems can significantly improve screening coverage, diagnostic timeliness, and inter-hospital coordination. However, to achieve national adoption and sustainability, Thailand must strengthen data interoperability, workforce capacity, and system governance. Building on these foundations will ensure that digital innovations translate into measurable, equitable, and enduring improvements in newborn and child health outcomes, in alignment with WHO and JCIH recommendations for universal EHDI [27,28].

This study has several limitations that should be considered when interpreting the findings. First, it was conducted in a single tertiary hospital, which may limit generalizability to other healthcare settings. The retrospective design relied on existing medical records, some of which contained incomplete or inconsistently documented data, particularly during the pre-database period. Although tracking improved for inborn newborns, loss-to-follow-up persisted and was largely driven by caregiver-related barriers such as limited awareness, transportation challenges, and work constraints, factors that the database alone cannot fully address. The post-integration assessment covered only 12 months, which may not adequately reflect long-term system performance or sustainability. Several methodological limitations also warrant attention. Diagnostic timing was dichotomized into ≤3 months versus >3 months due to the absence of uniformly recorded continuous time-to-diagnosis data, resulting in loss of granularity. Multiple comparisons across screening and diagnostic indicators may have increased the potential for Type I error. The absence of a control group restricts causal inference, as improvements may reflect temporal trends, increased staff experience, or concurrent changes in national screening algorithms. Additionally, multivariate analysis could not be performed because individual-level covariates were incomplete or inconsistently recorded; therefore, the results represent unadjusted associations. The database incorporated functions for tracking post-diagnostic intervention; however, timely hearing-aid fitting remained limited by caregiver non-adherence rather than system capacity. Despite automated reminders and follow-up calls, several caregivers did not return for scheduled appointments, resulting in amplification beyond six months in both periods. These observations highlight the need for enhanced family education, counseling, and community outreach to ensure that early detection leads to timely habilitation. Future research should include multicenter studies to evaluate generalizability, longitudinal assessments to examine long-term sustainability, and integration of multivariate or time-series analyses to identify independent predictors of screening and diagnostic outcomes. Evaluating cost-effectiveness, system usability, and interoperability with national health information platforms will also be essential to support large-scale implementation. Strengthening family engagement strategies, exploring mHealth or tele-audiology solutions, and addressing socioeconomic barriers will be critical to translating earlier diagnosis into timely intervention and improved developmental outcomes.

## 5. Conclusions

The integration of a dedicated NHS database improved screening coverage, referral accuracy, and diagnostic timeliness by strengthening workflow coordination, standardizing data entry, and enabling automated follow-up. However, modest gains in follow-up completion and persistently low hearing-aid uptake indicate that digital workflow enhancements alone cannot overcome caregiver-level socioeconomic barriers or systemic limitations in early intervention services. Because the study used a before–after design without a control group, the observed improvements should be interpreted as associations rather than causal effects. Tele-audiology solutions, including remote ABR interpretation and virtual counseling, offer promising opportunities to expand access in underserved regions and complement database-driven improvements. Sustaining these digital innovations will require national investment in infrastructure, cybersecurity, and interoperability, supported by coordinated planning between hospitals, academic partners, and the Ministry of Public Health. A comprehensive policy framework emphasizing workforce development and continuous quality improvement is essential to translate digital integration into long-term national impact. Future multicenter and longitudinal studies are needed to evaluate generalizability and identify independent predictors of improved outcomes.

## Data Availability

The datasets used and/or analyzed during the current study are available from the corresponding author upon reasonable request.

## Abbreviations

ABR	Auditory brainstem response
EHDI	Early hearing detection and intervention
LMICs	Low- and middle-income countries
NHS	Newborn hearing screening
TEOAE	Transient evoked otoacoustic emissions
UNHS	Universal newborn hearing screening
